# Supplementary material for: Efficacy and Safety of Low-Dose Cyclosporine Relative to Immunomodulatory Drugs Used in Atopic Dermatitis: A Systematic Review and Meta-Analysis
Source: J Clin Med. 2023 Feb 9;12(4):1390. doi: 10.3390/jcm12041390 (PMC9959975; doi:10.3390/jcm12041390)

Supplementary Table S1. Search strategies for database searching.

Search strategies for database searching

PubMed, EMBASE, and Cochrane library, Clinical Trial Registry, and World Health Organization International Clinical Trials Registry Platform were searched using separate comprehensive search strategies.

Search strategies

PubMed

Number	Search query	Results
1	"eczema*"[Title/Abstract] OR "atopic dermati*"[Title/Abstract] OR "dermati*"[Title/Abstract] OR "neurodermat*"[Title/Abstract] OR "prurigo"[Title/Abstract] OR "atopy"[Title/Abstract]	89,830
2	"eczema"[MeSH Terms] OR "dermatitis, atopic"[MeSH Terms] OR "dermatitis"[MeSH Terms] OR "neurodermatitis"[MeSH Terms] OR "prurigo"[MeSH Terms]	108,180
3	#1 OR #2	144,127
4	cyclosporins[Title/Abstract] OR sandimmune[Title/Abstract] OR sandimmun*[Title/Abstract] OR neoral[Title/Abstract] OR cyclosporine[Title/Abstract] OR cyclosporin*[Title/Abstract] OR ciclosporin*[Title/Abstract] OR "sandimmun neoral"[Title/Abstract] OR "OL 27400"[Title/Abstract] OR "cyclosporine A"[Title/Abstract] OR "cyclosporin A"[Title/Abstract] OR "CsA Neoral"[Title/Abstract] OR "CyA NOF"[Title/Abstract] OR "OL 27 400"[Title/Abstract] OR CsANeoral[Title/Abstract] OR "Cs A"[Title/Abstract] OR "Cy A"[Title/Abstract] OR CsA[Title/Abstract] OR CyA[Title/Abstract]	66,315
5	Cyclosporins[MeSH Terms] OR Cyclosporine[MeSH Terms]	39,799
6	#4 OR #5	73,696
7	#3 AND #6	1,263
8	Animals[MeSH Terms]	24,319,735
9	Humans[MeSH Terms]	19,464,322
10	#8 NOT #9	4,855,413
11	#7 NOT #10	1,046
12	#7 NOT #10 AND (randomizedcontrolledtrial[Filter])	32

Embase

Number	Search query	Results
1	eczema*:ab,ti OR 'atopic dermati*':ab,ti OR dermati*:ab,ti OR neurodermat*:ab,ti OR prurigo:ab,ti OR atopy:ab,ti	125,149
2	'eczema'/exp OR 'atopic dermatitis'/exp OR 'dermatitis'/exp OR 'neurodermatitis'/exp OR 'prurigo'/exp OR 'atopy'/exp	202,661
3	#1 OR #2	225,381
4	cyclosporins:ab,ti OR sandimmune:ab,ti OR sandimmun*:ab,ti OR neoral:ab,ti OR cyclosporine:ab,ti OR cyclosporin*:ab,ti OR ciclosporin*:ab,ti OR 'sandimmun neoral':ab,ti OR 'ol 27400':ab,ti OR 'cyclosporine a':ab,ti OR 'cyclosporin a':ab,ti OR 'csa neoral':ab,ti OR 'cya nof':ab,ti OR 'ol 27 400':ab,ti OR csaneoral:ab,ti OR 'cs a':ab,ti OR 'cy a':ab,ti OR csa:ab,ti OR cya:ab,ti	93,539
5	'cyclosporin derivative'/exp OR 'cyclosporine'/exp	158,090
6	#4 OR #5	184,058
7	#3 AND #6	6,208
8	'animal'/exp	29,198,920
9	'human'/exp	23,567,443
10	#8 NOT #9	5,631,477

11	#7 NOT #10	5,918
12	#11 AND 'randomized controlled trial'/de	127

Cochrane

Number	Search query	Results
1	Eczema*:ab,ti OR (atopic next dermati*):ab,ti OR dermati*:ab,ti OR neurodermat*:ab,ti OR prurigo:ab,ti OR atopy:ab,ti	9,107
2	[mh “Eczema”] OR [mh “Dermatitis, Atopic”] OR [mh “Dermatitis”] OR [mh “Neurodermatitis”] OR [mh “Prurigo”]	4,232
3	#1 OR #2	10,477
4	Cyclosporins:ab,ti OR sandimmune:ab,ti OR sandimmun*:ab,ti OR neoral:ab,ti OR cyclosporine:ab,ti OR cyclosporin*:ab,ti OR ciclosporin*:ab,ti OR "sandimmun neoral":ab,ti OR "OL 27400":ab,ti OR "cyclosporine A":ab,ti OR "cyclosporin A":ab,ti OR "CsA Neoral":ab,ti OR "CyA NOF":ab,ti OR "OL 27 400":ab,ti OR CsANeoral:ab,ti OR "Cs A":ab,ti OR "Cy A":ab,ti OR CsA:ab,ti OR CyA:ab,ti	8,483
5	[mh “Cyclosporins”] OR [mh “Cyclosporine”]	3,197
6	#4 OR #5	8,888
7	#3 AND #6	152
8	MeSH descriptor: [Animals] explode all trees	609,930
9	MeSH descriptor: [Humans] explode all trees	609,871
10	#8 NOT #9	59
11	#7 NOT #10	152
12	# 11 AND “Randomized Controlled Trial”:pt	33

Clinical Trial Registry

Number	Search query	Results
1	(eczema OR "atopic dermatitis" OR dermatitis OR neurodermatitis OR prurigo OR atopy) AND (cyclosporins OR sandimmune OR sandimmun* OR neoral OR cyclosporine OR cyclosporin* OR ciclosporin* OR "sandimmun neoral" OR "OL 27400" OR "cyclosporine A" OR "cyclosporin A" OR "CsA Neoral" OR "CyA NOF" OR "OL 27 400" OR CsANeoral OR "Cs A" OR Cy A" OR CsA OR CyA)	31

World Health Organization International Clinical Trials Registry Platform

Number	Search query	Results
1	(eczema OR "atopic dermatitis" OR dermatitis OR neurodermatitis OR prurigo OR atopy) AND (cyclosporins OR sandimmune OR sandimmun* OR neoral OR cyclosporine OR cyclosporin* OR ciclosporin* OR "sandimmun neoral" OR "OL 27400" OR "cyclosporine A" OR "cyclosporin A" OR "CsA Neoral" OR "CyA NOF" OR "OL 27 400" OR CsANeoral OR "Cs A" OR Cy A" OR CsA OR CyA)	32

Supplementary Figure S1. Sensitivity analysis of the adverse events.

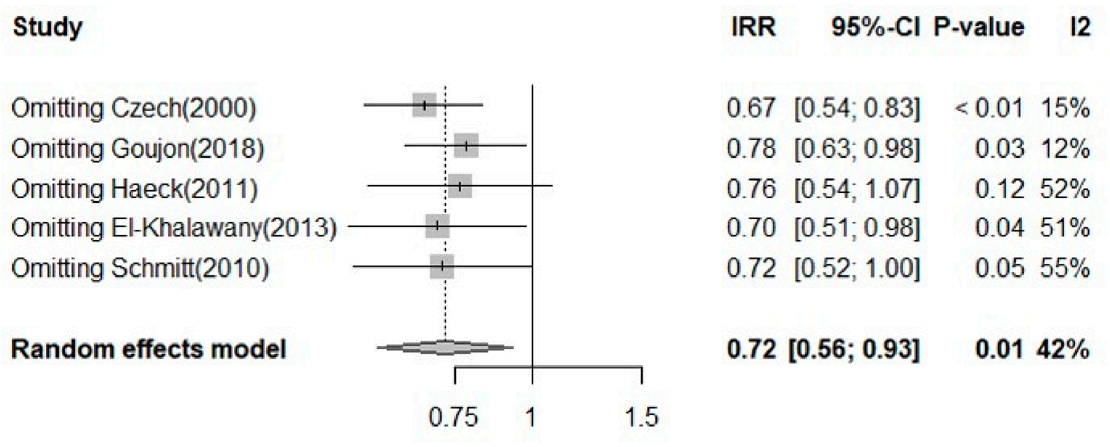

Supplement: Supplementary file 1 [file jcm-12-01390-s001.zip › jcm-2157767-supplementary.pdf]
